# The Role of Gut Microbiota in Liver Regeneration After Partial Hepatectomy: New Evidence From Animal and Human Studies

**DOI:** 10.1096/fj.202505079R

**Published:** 2026-07-09

**Authors:** Roberto Loi, Gabriella Simbula, Andrea Caddeo, Monica Pibiri

**Affiliations:** ^1^ Department of Biomedical Sciences, Oncology and Molecular Pathology Unit University of Cagliari, Cittadella Universitaria di Monserrato Cagliari Italy

**Keywords:** gut microbiota, gut–liver axis, liver regeneration, liver transplantation, partial hepatectomy

## Abstract

Liver regeneration is increasingly recognized as a process influenced not only by hepatocellular signaling but also by the gut–liver axis, where gut microbiota‐derived metabolites, immune mediators, and extracellular vesicles modulate hepatic recovery after liver damage. In this review, we explore recent progress in understanding the gut microbiota's role in liver regeneration and discuss its therapeutic potential in the context of hepatic surgery and liver transplantation. Emerging evidence shows that beneficial microbial taxa, including *Akkermansia muciniphila, Bifidobacterium longum*, and *Parabacteroides distasonis* , enhance liver regeneration by regulating short‐chain fatty acid production, bile acid metabolism, and tricarboxylic acid cycle pathways, while dysbiosis and microbial translocation can impair regenerative outcomes. Key host‐microbiome interactions, particularly the Farnesoid X Receptor (FXR)‐Fibroblast Growth Factor 19 (FGF19) signaling axis, play a central role in protecting hepatocytes from bile acid overload and supporting regeneration, highlighting the therapeutic potential of FXR agonists, FGF19 mimetics, probiotics, dietary interventions, and metabolite supplementation. At the same time, monitoring bile acids profiles alongside gut microbiota composition may allow early detection and prevention of complications. In addition, microbial‐derived markers such as the lipopolysaccharide/lipoteichoic acid ratio may serve as predictive biomarkers for post‐hepatectomy liver failure. Adjunctive approaches, including vitamin D supplementation, may further support regeneration through vitamin D receptor‐mediated regulation of bile acid homeostasis and cell‐cycle progression. In the context of live donor liver transplantation, the detection of occult bacteremia further underscores the complexity of host‐microbiome interactions and suggests that microbiological surveillance could improve postoperative management. Collectively, these findings emphasize the importance of microbiota‐targeted strategies to improve hepatic regeneration, reduce postoperative complications, and optimize outcomes following liver surgery and transplantation.

## Graphical abstract

Partial hepatectomy (PH) reshapes gut microbiota composition, resulting in the release of microbial metabolites and components, including short chain fatty acids (SCFAs)/β‐hydroxybutyric acid (BHB), bacterial DNA and extracellular vesicles (EVs), bile acids (deoxycholic acid, DCA), and aminobenzoate degradation products. These microbiota‐derived signals are transported to the liver through the portal circulation, where they regulate inflammation, metabolism, cell cycle signaling, and hormonal pathways to support liver regeneration. Conversely, dysbiosis, impaired bile acid (BA)/Fibroblast growth factor 19 (FGF19) signaling, and vitamin D deficiency may lead to impaired LR.

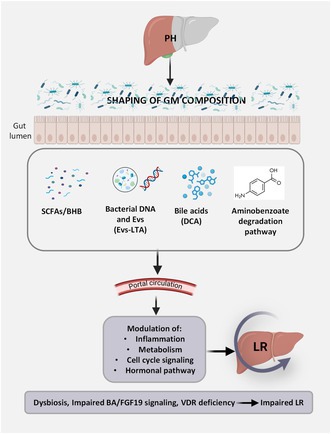

**Abbreviations**
2/3 PH: two‐thirds partial hepatectomy
5‐HT: 5‐hydroxytryptamine
Abx: antibiotic
AhR: aryl hydrocarbon receptor
AKT: protein kinase B
ALT: alanine aminotransferases

*A. muciniphila*

:
*Akkermansia muciniphila*

ASBT: sodium‐dependent bile acid transporter
BAs: bile acids
BHB: β‐hydroxybutyric acid
*BL*
:
*Bifidobacterium longum*

BMI: body mass index
BSEP: bile salt export pump
BSH: bile salt hydrolase
BT: bacterial translocation
C4: 7α‐hydroxy‐4‐cholesten‐3‐one
CA: cholic acid
CDCA: chenodeoxycholic acid
CD‐HFD: choline‐deficient high‐fat diet
CRLM controls: colorectal liver metastasis controls
CYP7A1: cholesterol 7α‐hydroxylase
DCA: deoxycholic acid
DDLT: deceased donor liver transplantation
DR: delayed recovery
ECM: extracellular matrix
EVs: extracellular vesicles
*F/B* ratio: *Firmicutes*‐to‐*Bacteroidetes* ratio
FGF: fibroblast growth factor
FGFR: fibroblast growth factor receptor
FMT: fecal microbiota transplantation
Foxm1: forkhead (FKH) box protein M1
FXR: farnesoid X receptor
GF mice: germ‐free mice
GLP‐1: glucagon‐like peptide‐1
GM: gut microbiota
GPRs: G‐protein‐coupled receptors
H_2_
: hydrogen
HCC: hepatocellular carcinoma
HFD: high‐fat diet
HGF: hepatocyte growth factor
HSCs: hepatic stellate cells
hVDR KO mice: hepatocyte‐specific vitamin D receptor knock‐out mice
IAA: indole‐3‐acetic acid
IL‐6: interleukin‐6
IPA: indole‐3‐propionic acid
LCA: lithocholic acid
LDLT: living donor liver transplantation
LFR: liver function recovery
LPS: lipopolysaccharide
LR: liver regeneration
LT: liver transplantation
LTA: lipoteichoic acid
MAFLD: metabolic dysfunction‐associated fatty liver disease
MDA: malondialdehyde
MELD: Model for End‐Stage Liver Disease
NAFLD: non‐alcoholic fatty liver disease
NF‐κB: nuclear factor kappa‐light‐chain‐enhancer of activated B cells
NKTs: natural killer T cells
NLR: nucleotide‐binding oligomerization domain‐like receptors
NR: normal recovery
OS: overall survival
PBAs: primary bile acids
pCCA: perihilar cholangiocarcinoma

*P. distasonis*

: *Parabacterioides distasonis*
PH: partial hepatectomy
PHLF: post‐hepatectomy liver failure
PI3K: phosphoinositide 3‐kinase
POAs: predominant obligate anaerobes
PPARγ: peroxisome proliferator‐activated receptor γ
PXR: pregnane X receptor
RT‐qPCR: reverse transcription quantitative polymerase chain reaction
RXR: retinoid X receptor
S1P: sphingosine‐1‐phosphate
SBAs: secondary bile acids
SCD1: stearoyl‐CoA desaturase 1
SCFAs: short‐chain fatty acids
SOD: superoxide dismutase
STAT3: signal transducer and activator of transcription 3
TCA cycle: tricarboxylic acid
TGR5: Takeda G protein‐coupled receptor 5
TLR4: toll‐like receptor 4
TNF‐α: tumor necrosis factor‐α
TRP: tryptophan
VDR: vitamin D receptor
XGF: ex‐germ‐free

## 1 Background

The liver has a remarkable ability to regenerate, which is an evolutionary adaptation to its constant exposure to dietary and environmental toxins. Although liver regeneration (LR) occurs in response to various injuries, the mechanisms and physiological context vary significantly depending on the injury type. Specifically, LR after partial hepatectomy (PH) shows distinct features compared to regeneration after viral or drug‐induced liver damage. Unlike the gradual and often diffuse changes typical of toxic or infectious injury, PH induces abrupt and profound alterations in hepatic physiology, including hemodynamic changes in portal vein flow and pressure, tissue ischemia/hypoxia, and hemostasis‐related platelet activation [1, 2, 3, 4, 5]. While some of these factors also occur in viral or drug‐induced liver injuries, their intensity and sudden onset after surgical resection are seen as primary triggers and driving forces for rapid liver regrowth. This regenerative capability has enabled increasingly extensive liver resections in clinical practice, with direct relevance to hepatic surgery and liver transplantation (LT). However, post‐hepatectomy liver failure (PHLF) remains a serious and often lethal complication [6], highlighting the urgent need for reliable predictive markers of regenerative outcomes following surgery.

To date, most research on LR has focused on intracellular signaling pathways within hepatocytes and other liver‐resident cells. However, emerging evidence points to the crucial role of the gut microbiota (GM) in modulating the liver regeneration response [7]. Over the past decade, it has become increasingly clear that the GM exerts a substantial influence on human disease. This effect is largely attributed to the intestinal mucosa, which both shelters the microbiota and functions as a selectively permeable barrier. Through this interface, microbial components and their vast array of metabolic products can translocate into the bloodstream [8]. In this context, the liver represents a particularly vulnerable organ, as it is the first to receive venous drainage from the alimentary tract via the portal circulation [9]. Consequently, it is continuously exposed to gut‐derived microbial products. Within the liver, the sinusoidal endothelium is relatively permeable, further facilitating the diffusion of microbial metabolites (and, under certain conditions, even microorganisms themselves) into hepatic tissue and the systemic circulation [10]. The gut–liver axis, through microbial metabolites, immune signaling, and barrier function, may profoundly influence the regenerative processes. Therefore, understanding how GM regulates LR at the molecular level is of growing interest, not only for its scientific implications but also for the potential development of novel therapeutic strategies to enhance liver growth when LR is impaired.

In this review, we explore the most recent studies, from January 2024 to December 2025, focusing on GM's influence in LR and discuss its therapeutic potential in the context of hepatic surgery and LT.

### 1.1 The GM

The GM is a complex and diverse community of bacteria and other microorganisms residing in the human gastrointestinal tract, forming the largest symbiotic ecosystem with the host [11]. It is estimated that the human gut harbors between 10^13^ and 10^14^ microorganisms [12]. The predominant bacterial phyla include *Firmicutes* (60%–80%), *Bacteroidetes* (20%–40%), along with *Proteobacteria, Actinobacteria, Verrucomicrobia, Fusobacteria*, and *Cyanobacteria* [13]. The relative abundance of these phyla significantly impacts human health, including LR [14, 15]. Due to its critical role in host metabolism and immune regulation, the GM has been increasingly referred to as a “virtual metabolic organ” or a “previously forgotten organ.” One of its main effects on liver health occurs via the gut–liver axis, an anatomical and functional bidirectional communication system between the gut and the liver [16].

The liver, through the bile ducts and systemic circulation, releases bile acids (BAs) and other bioactive molecules into the intestine, where they interact with the GM [17]. Conversely, microbial and host‐derived metabolites, along with exogenous compounds from the gut, reach the liver via the portal vein, affecting hepatic function.

A key component of the gut–liver axis is the intestinal barrier, which includes the epithelial layer of the intestinal mucosa and the intercellular junctions that control selective permeability. This barrier limits the translocation of microbes and harmful metabolites while allowing the active transport of nutrients [18]. Tight junctions, adherens junctions, and desmosomes maintain the integrity of this barrier [19]. Additional contributors include mucins, antibacterial peptides, immunoglobulins, and intraepithelial lymphocytes, which further support barrier function [17, 20]. When intestinal permeability increases, as seen in cases of dysbiosis or inflammation, microbial translocation is enhanced, leading to liver damage and impaired LR [19, 21]. Inflammatory processes in the gut, often resulting from microbiota imbalance, have been documented in several liver diseases.

**FIGURE 1:**
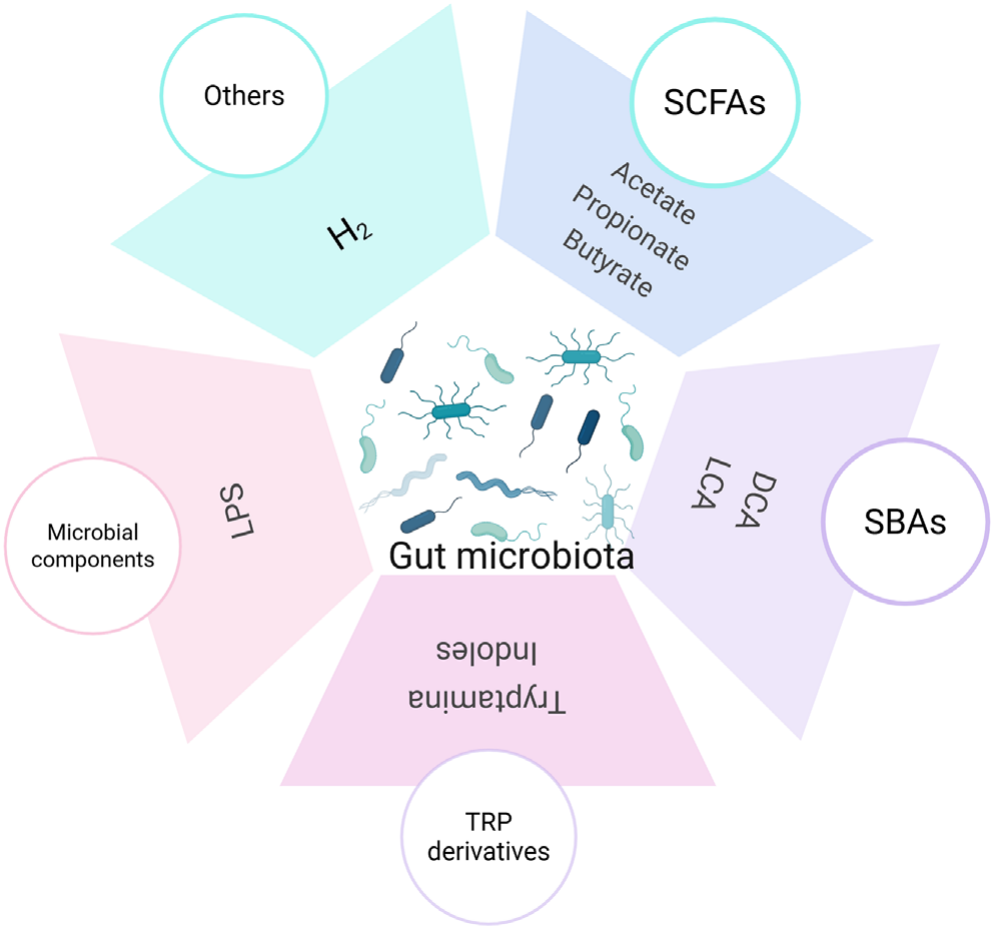
GM‐derived products involved in LR. Key GM‐derived metabolites/components transported via the portal vein to the liver include SCFAs, products of bacterial TRP fermentation, including indoles and derivatives, gut bacteria‐produced H_2_, SBAs, and LPS. GM, gut microbiota; LPS, lipopolysaccharide; LR, liver regeneration; SBAs, secondary bile acids; SCFAs, short‐chain fatty acids; TRP, tryptophan. This figure was created in BioRender.

The GM and its derived products regulate both metabolism and cytokine secretion in gut and liver cells, thereby directly influencing LR [21, 22]. The two main ways by which the GM promotes LR are through microbial products, as well as immune modulation. Key microbial products transported via the portal vein include true metabolites, such as short‐chain fatty acids (SCFAs), products of bacterial tryptophan (TRP) fermentation, including indoles, their derivatives, and gut bacterial‐produced H_2_; co‐metabolites, such as secondary bile acids (SBAs), and microbial components, such as lipopolysaccharide (LPS) (Figure 1).

### 1.2 The Role of GM in LR: Insights From Experimental Studies

#### 1.2.1 Changes in GM Composition During LR

Previous studies have demonstrated that LR is delayed following 2/3 PH in germ‐free or LPS‐resistant rodents [23]. Consistently, Wu et al. [24] showed that depletion of gut bacteria by oral ampicillin treatment impaired LR in mice by disrupting hepatic innate immune tolerance and triggering hyperactivation of hepatic natural killer T cells (NKTs), which impede LR. In the same year, Liu et al. [15] investigated changes in GM composition during LR after 2/3 PH in mice. They observed an inverse correlation between the two most abundant bacterial phyla, *Bacteroidetes* and *Firmicutes*, with *Bacteroidetes* steadily increasing and *Firmicutes* decreasing during LR. At the family level, this expansion of *Bacteroidetes* was mainly due to increases in *S24‐7* (former name for the bacterial family *Muribaculaceae*) and *Rikenellaceae*, while the decrease in *Firmicutes* was driven by reductions in families between *Clostridiales*, including *Lachnospiraceae* and *Ruminococcaceae*. These consistent compositional shifts were observed over 9 days post‐surgery, spanning the priming, proliferative, and termination phases of LR [25]. Using RNA sequencing, six unique hepatic gene expression patterns were identified during LR. Notably, Spearman correlation analysis linked the populations of *Clostridiales* families, *Lachnospiraceae* and *Ruminococcaceae*, to a common hepatic gene expression profile, suggesting their involvement in similar biological processes during LR, whereas *S24‐7* from *Bacteroidetes* showed an opposing pattern. Furthermore, representatives of the *Ruminococcaceae, Lachnospiraceae*, and *S24‐7* families were closely associated with opposing correlation patterns with genes involved in host metabolism (oxidative phosphorylation, mitochondrial function, tricarboxylic acid, TCA, cycle) and immune responses (toll‐like receptor 4, *Tlr4*, nuclear factor kappa‐light‐chain‐enhancer of activated B cells, *NF‐κB*, fibroblast growth factor receptor 1 and 4, *Fgfr1* and *Fgfr4*, *Cd44*, and *Cd86*). This interplay likely contributes to the fine‐tuning of immune‐metabolic interactions, promoting a homeostatic state favorable for tissue regeneration [15].

In 2019, Bao et al. [26] performed a similar analysis on rats undergoing 2/3 PH or sham surgery. Their results revealed a time‐dependent fluctuation in the *Firmicutes*‐to‐*Bacteroidetes* (*F/B*) ratio during LR, with increases during early (12–24) and late (3–14 days) phases and a decrease during the middle phase (30–48 h). Within *Firmicutes*, the most notable change during 12–24 h was an increase in *Clostridiales* families, including *Ruminococcaceae* and *Lachnospiraceae*, known for their substantial contribution to endogenous butyrate production, a key molecule for gut health and energy extraction from food [27, 28]. This shift in microbial community and increased *F/B* ratio early after 2/3 PH likely supports enhanced energy extraction to fuel hepatic regeneration. However, previous studies have also shown that lipid overload impairs LR [29, 30] and that a decreased *F/B* ratio may accelerate LR [31]. Consistently, Bao et al. [26] found that during the active proliferative phase (30–48 h), fecal samples of hepatectomized animals showed an increase in *Bacteroidetes* and *Proteobacteria* with a relative reduction in *Firmicutes* compared to earlier and later phases. These results suggest that microbial shifts during LR adaptively meet changing demands for cell proliferation and energy metabolism. Functional analysis revealed that bacteria enriched in the first 12 h after PH primarily have metabolic functions related to carbohydrate, glycan, and lipid metabolism, while at 48 h bacterial populations producing metabolites associated with cell proliferation (cofactors, vitamins, nucleotides, nicotinate, and nicotinamide) predominate. These functional shifts in the microbiota likely facilitate LR progression. Importantly, Bao et al. [26] also evaluated the impact of GM modulation. Rats treated with oral antibiotics (Abx) for 4 weeks prior to 2/3 PH exhibited significantly reduced LR, while subsequent fecal microbiota transplantation (FMT) restored regenerative capacity. These findings underscore the co‐regulatory role of GM in metabolic and proliferative processes during LR [26].

#### 1.2.2 Mechanisms Involved in LR Modulation by GM Metabolites

**FIGURE 2:**
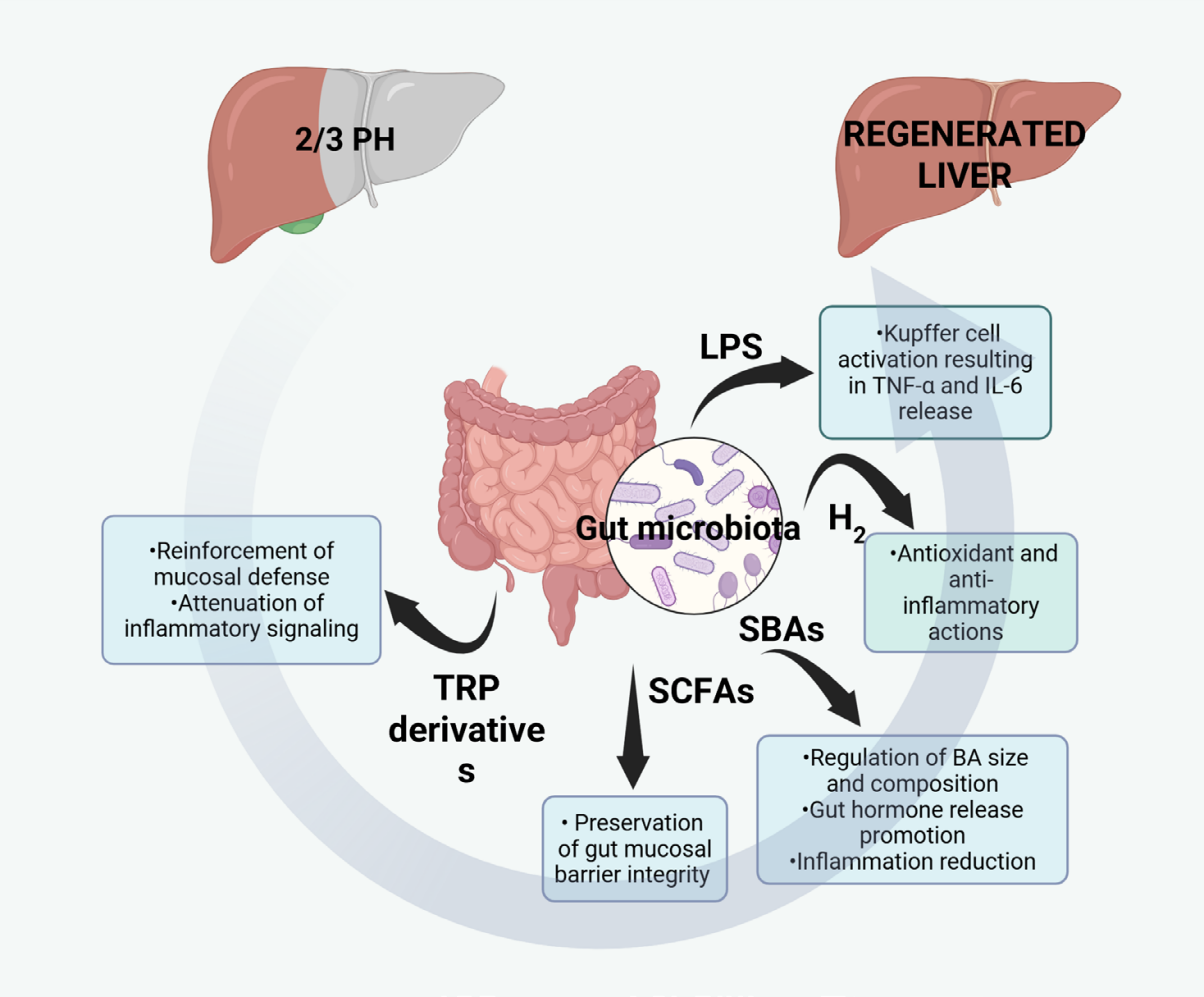
Effects of GM‐derived products on LR. GM metabolites regulate both metabolism and cytokine secretion in gut and liver cells, thereby directly influencing LR: (i) SCFAs support LR by maintaining gut mucosal barrier integrity through modulation of cell adhesion and immune response modulation; (ii) SBA‐mediated TGR5 activation enhances LR by regulating hepatic inflammatory responses, limiting hepatocyte necrosis after PH, promoting gut hormone release and optimizing BA pool composition; (iii) controlled levels of LPS produced by Gram‐negative bacteria after surgery can stimulate IL‐6 and TNF‐α secretion from Kupffer cells and promote LR; (iv) TRP derivatives enhance LR by improving intestinal barrier integrity and reducing gut permeability; and (v) H_2_ endogenously produced by GM bacteria promotes LR by exerting antioxidant and anti‐inflammatory effects. GM, gut microbiota; IL‐6, interleukin 6; LPS, lipopolysaccharide; LR, liver regeneration; SBAs, secondary bile acids; SCFAs, short‐chain fatty acids; TNF‐α, tumor necrosis factor α; TRP, tryptophan. This figure was created in BioRender.

GM‐derived products, namely gut‐derived LPS, H_2_, SBAs, SCFAs, and products of bacterial TRP fermentation, have been shown to stimulate LR through several mechanisms (Figure 2).

LPS is the major component of the outer wall of the cytoderm of Gram‐negative bacteria. There is a large number of Gram‐negative bacteria in the human gut, such as *Escherichia coli* , *Proteus*, and *Pseudomonas aeruginosa* . When these bacteria die, LPS will come off from dissolving bacterial and exert its toxicity by acting on human cells. Just as BAs, the right amount of LPS is beneficial to LR [32]. LPS promotes LR by inducing tumor necrosis factor‐α (TNF‐α) and interleukin 6 (IL‐6) release from Kupffer cells via TLR4 signaling following 2/3 PH [33]. However, excess LPS exacerbates inflammation and liver damage in acute injury models such as paracetamol overdose, and chronic LPS elevation may lead to excessive TLR4 activation, hepatocyte hyperproliferation, and hepatocellular carcinoma (HCC) development [34]. Thus, gut‐derived LPS exerts a dual role in liver injury and regeneration depending on exposure intensity and duration. In particular, GM dysbiosis downregulates the synthesis of the farnesoid X receptor (FXR) and the Takeda G protein‐coupled receptor 5 (TGR5) in the ileum [16, 35, 36], inducing bacterial translocation and bacterial overgrowth, particularly of gram‐negative organisms, and perpetuating increased gut permeability [37]. This contributes to elevated levels of LPS, which can activate NF‐κB [38] through TLRs and nucleotide‐binding oligomerization domain‐like receptors (NLRs). These pathways trigger the production of inflammatory cytokines and chemokines that enter the portal circulation, promoting hepatic inflammation and contributing to liver disease progression [39, 40]. In this context, pharmacological modulation of the GM by using probiotics, prebiotics, or selective antibiotics to control LPS levels represents a promising therapeutic approach [34].

Hydrogen (H_2_) gas, produced endogenously by gut bacteria, also contributes to LR (Figure 1). Yu et al. [41] showed that lactulose accelerated LR in rats after 2/3 PH by inducing endogenous H_2_ production, which exerts antioxidant and anti‐inflammatory effects, evidenced by increased hepatic superoxide dismutase (SOD) activity and reduced malondialdehyde (MDA), IL‐6, and TNF‐α levels. Supplementation with exogenous H_2_ (via H_2_‐rich saline) mimicked lactulose's protective effects, while antibiotics suppressed the regenerative benefit of lactulose by reducing H_2_ production. Given that H_2_ levels depend on the balance between H_2_‐producing (e.g., *Bacteroides, Firmicutes*) and H_2_‐consuming bacteria, these findings implicate gut dysbiosis‐induced H_2_ depletion as a mechanism impairing LR [41, 42].

SBAs. BAs are critical metabolic signals tightly regulated through bidirectional interactions between the host and microbiota via enterohepatic circulation. Primary BAs (PBAs), mainly cholic acid (CA) and chenodeoxycholic acid (CDCA), are synthesized from cholesterol in the liver through the classical pathway initiated by cholesterol 7α‐hydroxylase (CYP7A1) [43] or the alternative pathway involving sterol 27‐hydroxylase (CYP27A1) [44], involving sterol 27‐hydroxylase (CYP27A1). PBAs are subsequently conjugated with glycine or taurine to form glyco‐ and tauro‐conjugated BAs that are more soluble and less cytotoxic [45]. These conjugated BAs are secreted into bile and released into the duodenum, where they facilitate lipid emulsification and absorption [46]. Approximately 95% of BAs are reabsorbed in the terminal ileum via the apical sodium‐dependent bile acid transporter (ASBT) and returned to the liver through the enterohepatic circulation [47]. The remaining fraction reaches the colon, where it undergoes extensive microbial biotransformation. The GM profoundly influences BA composition by deconjugating PBAs and converting them into SBAs, including deoxycholic acid (DCA) and lithocholic acid (LCA), while also generating BA isomers and other metabolites through dehydroxylation, dehydration, and sulfation reactions [47, 48]. Deconjugation occurs via bile salt hydrolases (BSHs) expressed by *Firmicutes, Bacteroidetes*, and *Actinobacteria*, leading to better reabsorption of BAs [49] while the production of LCA and DCA depends on 7α‐dehydroxylation by *Clostridium* and *Eubacterium* [34, 50]. CA, CDCA, and DCA are reabsorbed and transported back to the liver [51] while a smaller fraction is excreted in feces and urine. BAs tightly regulate their own synthesis through activation of FXR, which suppresses hepatic BA production via negative feedback [47]. Consequently, efficient intestinal BA reabsorption inhibits de novo synthesis, whereas fecal BA loss stimulates compensatory hepatic production. Beyond their digestive functions, both PBAs and SBAs act as signaling molecules through FXR and TGR5 [52, 53]. These receptors regulate lipid, glucose, steroid, xenobiotic, and energy metabolism [54, 55, 56, 57], and multiple studies have demonstrated that the GM modulates BA composition and host metabolism in an FXR‐dependent manner [48, 58]. FXR activation in hepatocytes promotes metabolic reprogramming and cell proliferation, whereas intestinal FXR induces FGF19 (FGF15 in rodents) production in ileal enterocytes. This gut–liver signaling axis further regulates hepatic metabolism and LR [59, 60, 61]. Following PH, disruption of enterohepatic circulation leads to transient systemic and intrahepatic BA overload [62, 63, 64]. Although elevated BA levels can cause hepatocyte injury because of their detergent properties, they also activate protective and proliferative signaling pathways. FXR and TGR5 are therefore essential for maintaining biliary homeostasis and supporting LR by modulating hepatocyte and cholangiocyte function [53]. Clinical evidence further highlights the importance of intestinal BA metabolism during LR. Patients undergoing major PH without external biliary drainage, which preserves BA passage into the intestine, exhibit improved LR compared with patients receiving biliary drainage [65, 66]. This suggests that interruption of enterohepatic BA circulation may deprive the regenerating liver of important pro‐regenerative signals [67]. GM‐dependent BA metabolism influences both the composition and size of the BA pool, thereby modulating liver injury and regeneration [34]. In particular, bacterial deconjugation enhances colonic BA absorption and increases the abundance of conjugated PBAs, triggering FXR‐mediated hepatoprotective and proliferative responses via FGF15/19 signaling, which suppresses CYP7A1, the rate‐limiting enzyme in BA synthesis. In addition, FGF15 directly promotes LR by stimulating hepatocyte and cholangiocyte proliferation through the FGF15‐FGFR4‐Signal transducer and activator of transcription 3 (STAT3)‐forkhead (FKH) box protein M1 (Foxm1) signaling axis [59, 68].

SBAs are also potent agonists of TGR5 [69]. Activation of TGR5 by GM‐derived SBAs enhances LR by modulating hepatic inflammation, reducing hepatocyte necrosis after PH, optimizing BA pool composition, and stimulating release of glucagon‐like peptide‐1 (GLP‐1) [34]. Collectively, these findings support a coordinated framework in which microbiota‐derived BAs regulate LR through interconnected FXR, TGR5, and FGF15/19 signaling pathways that converge on hepatocyte proliferation and tissue repair.

SCFAs are major metabolites produced by GM fermentation of carbohydrates and proteins. SCFAs have demonstrated beneficial effects in regulating immunity and metabolism during liver diseases and regeneration [70, 71]. Acetate and propionate are primarily produced by *Bacteroidetes*, while butyrate derives mainly from *Firmicutes*; together, they comprise approximately 95% of SCFAs [34]. SCFAs support LR by maintaining gut mucosal barrier integrity and enhancing liver metabolic homeostasis. They serve as energy substrates for colonocytes, stimulate mucin and mucosal lipid synthesis, and strengthen tight junctions [72, 73, 74, 75]. SCFAs also modulate immune responses by promoting differentiation of regulatory T cells into anti‐inflammatory phenotypes via G‐protein‐coupled receptors (GPRs) and histone deacetylase inhibition [76, 77, 78, 79]. This preserves the mucosal barrier, limiting bacterial and LPS translocation that facilitates LR [32]. Moreover, activation of specific GPRs can directly stimulate LR‐associated signaling pathways [32]. Metabolically, SCFAs reaching the liver via the portal vein regulate glucose and lipid homeostasis through peroxisome proliferator‐activated receptor γ (PPAR‐γ)‐dependent mechanisms [80] and enhance postprandial secretion of gut hormones like GLP‐1 and peptide YY, further improving metabolic profiles [32, 81, 82]. Yin et al. [83] underscored the indispensable role of SCFAs in LR by showing that antibiotic‐treated mice undergoing 2/3 PH exhibited dysbiosis with a shift from *Firmicutes* and *Bacteroidetes* to *Proteobacteria* dominance, associated with poor survival and delayed hepatocyte proliferation. Acetate produced by SCFA‐generating *Firmicutes* and *Bacteroidetes* is essential for LR and overall lipid metabolism, especially for membrane lipid biosynthesis. This is corroborated by human liver biopsy data demonstrating upregulation of lipogenic enzymes during regeneration. The lipogenic enzyme stearoyl‐CoA desaturase (SCD1) emerges as a key regulator of SCFA metabolism, linking microbial composition, lipid metabolism, and LR [83].

Products of bacterial TRP fermentation, including indoles, such as skatole and tryptamine, have been shown to reinforce mucosal defense and reduce intestinal permeability [84]. They also exert protective effects in the liver by attenuating inflammatory signaling, partly through modulation of the NF‐κB pathway [70]. Indole itself promotes the release of GLP‐1 from colonic L cells, which contributes to maintaining glucose homeostasis and thus indirectly supports hepatic metabolic stability [85]. Derivatives of indole, such as indole‐3‐acetic acid (IAA) and indole‐3‐propionic acid (IPA), act as endogenous ligands of the aryl hydrocarbon receptor (AhR). Activation of this pathway strengthens intestinal barrier integrity and dampens inflammatory responses in both macrophages and hepatocytes, thereby protecting the liver from injury [86]. IPA, in particular, also serves as an activator of the pregnane X receptor (PXR), further enhancing epithelial barrier function, reducing the translocation of intestinal LPS, and supporting regenerative processes after hepatic injury [87].

#### 1.2.3 Interactions Between the Extracellular Matrix (ECM) and the GM in LR

Amin and colleagues examined LR and the hepatic matrisome following 2/3 PH in germ‐free (GF) mice, GF mice reconstituted with normal GM (XGF), and conventionally raised controls [88]. They observed that regenerative capacity was markedly impaired in GF animals, with reduced hepatocellular proliferation at 72 h post‐surgery. This defect was preceded by an early reduction in the expression of cytokine receptor genes and the hepatocyte growth factor (*Hgf*) gene just 3 h post‐PH. Notably, restoration of the GM in XGF mice re‐established a regenerative response comparable to that of control animals. Gene‐expression analyses further revealed that GF mice exhibited significantly fewer differentially expressed matrisome‐associated genes compared with XGF mice at both the early (3 h) and later (72 h) post‐surgery time points, suggesting that the interaction between the GM and the matrisome is crucial for orchestrating hepatic remodeling during regeneration.

Taken together, these findings suggest that the GM influences LR in multiple ways, mainly through the production of bioactive metabolites that regulate systemic inflammation, immune responses, energy metabolism, barrier integrity, and hepatocellular signaling. Despite these advances, the complex interactions between microbial communities, their metabolites, and hepatic repair mechanisms are still only partially understood and remain an important area of ongoing research. Understanding these complex gut–liver axis interactions opens new avenues for therapeutic strategies to improve liver repair and recovery. In recent years, several studies conducted in both experimental animal models and humans have aimed to elucidate the contribution of the GM to LR following PH and in the context of LT. The main studies published over the past 2 years are summarized and discussed below.

## 2 New Evidence

### 2.1 GM and LR After 2/3 PH: Animal Studies

**FIGURE 3:**
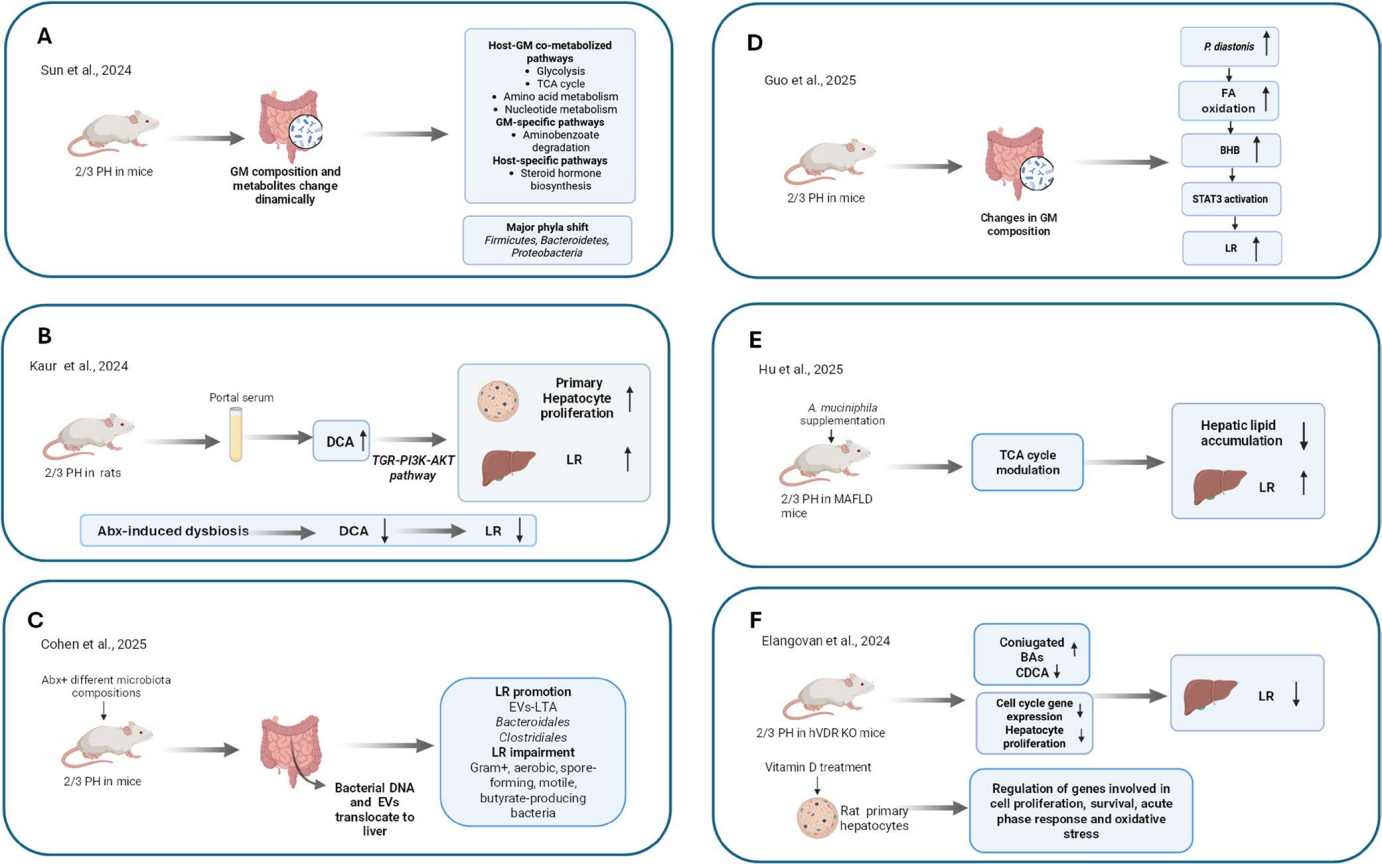
GM and LR after 2/3 PH: Animal studies. Summary of latest animal studies on the regulation of LR by the GM. (A) Sun et al. [89]: GM composition and derived metabolites change dynamically during LR. PH reshapes both host‐microbe co‐metabolic pathways, such as glycolysis, the TCA cycle, and amino acid and nucleotide metabolism, and host‐specific pathways, notably steroid hormone biosynthesis. Concurrently, major bacterial phyla (*Firmicutes, Bacteroidetes*, and *Proteobacteria*) shift, with aminobenzoate degradation emerging as a key GM‐associated pathway. GM, gut microbiota; LR, liver regeneration; PH, partial hepatectomy; TCA, tricarboxylic acid. (B) Kaur et al. [90]: DCA in portal blood promotes LR. Metabolomic analysis identified several changes between controls and PH, and between Abx‐treated PH and PH in portal serum. In particular, DCA was increased in portal serum of PH rats and decreased in Abx + PH rats. In vitro, DCA enhanced hepatocyte proliferation via the TGR5‐PI3K‐AKT pathway, linking microbial metabolism to LR signaling. These findings highlight a key role for portal DCA in promoting LR. Abx, antibiotic; AKT, protein kinase B; DCA, chenodeoxycholic acid; LR, liver regeneration; PH, partial hepatectomy; PI3K, phosphoinositide 3‐kinase; TGR5, Takeda G protein‐coupled receptor. (C) Cohen et al. [91]: bacterial DNA and EVs can translocate to the liver after surgery and influence LR. LR is negatively associated with Gram‐positive, aerobic, motile, spore‐forming, and butyrate‐producing bacteria, particularly in dysbiotic or obese GM. In contrast, EVs‐LTA and commensal taxa such as *Bacteroidales* and *Clostridiales* are linked to improved recovery. EVs, extracellular vesicles; GM, gut microbiota; LR, liver regeneration; LTA, lipoteichoic acid; (D) Guo et al. [92]: after PH, changes in GM composition were observed. In particular, *P. distasonis* correlated with hepatocyte proliferation. Mechanistically, *P. distasonis* increased hepatic FA oxidation and BHB production, which drove LR through STAT3 signaling activation. BHB, β‐hydroxybutyric acid; FA, fatty acid; LR, liver regeneration; *P. distasonis*, *Parabacterioides distasonis*; PH, partial hepatectomy; STAT3, signal transducer and activator of transcription 3. (E) Hu et al. [93]: MAFLD impairs LR after PH, alongside marked changes in GM and liver metabolites over time. Supplementation with *A. muciniphila* reduced hepatic lipid accumulation and enhanced LR in MAFLD mice, likely by modulating the TCA cycle. *A. muciniphila* , *Akkermansia muciniphila* ; GM, gut microbiota; LR, liver regeneration; MAFLD, metabolic dysfunction‐associated fatty liver disease; PH, partial hepatectomy; TCA, tricarboxylic acid. (F) Elangovan et al. [94]: after PH, hVDR KO mice exhibited impaired LR and reduced hepatocyte proliferation, accompanied by marked increases in conjugated BAs and decreased CDCA levels compared with control animals. These findings are consistent with a failure to restore normal hepatic function following surgery. RT‐PCR analysis of hVDR KO and control livers revealed significant alterations in the expression of cell cycle‐related genes. In addition, gene expression profiling of vitamin D‐treated hepatocytes demonstrated regulation of pathways involved in liver proliferation, hepatitis, liver hyperplasia/hyperproliferation, and liver necrosis/cell death. BAs, bile acids; CDCA, chenodeoxycholic acid; hVDR KO mice, hepatocyte‐specific vitamin D receptor‐knock out mice; LR, liver regeneration; PH, partial hepatectomy; RT‐PCR, reverse transcription polymerase chain reaction.

Results from experimental studies are summarized in Figure 3.

#### 2.1.1 GM and Metabolic Signaling Involved in LR

Advances in metabolomics and bioinformatics have revealed associations between the functional activity of the GM, alterations in endogenous metabolites, and LR. Nevertheless, the specific contribution of metabolites directly derived from the GM to LR has yet to be fully elucidated. To improve the understanding of gut–liver axis interaction involving GM in LR Sun et al. [89] in a recent study analyzed the metabolites present in the intestinal contents of mice at various time points following 2/3 PH using a metabolomics‐based approach (Figure 3A). The data obtained showed significant changes in GM composition at different stages of LR, particularly at 6‐ and 36‐h post‐surgery. According to previous data [15] the α and β diversity of the gut bacteria both changed. Specifically, 2/3 PH led to a decrease in phyla belonging to *Firmicutes* and *Verrucomicrobia* and an increase in phyla from *Bacteroidetes* and *Proteobacteria*. At the family level, there was a reduction in *Akkermansiaceae* and *Lactobacillaceae* (*Firmicutes*), along with an increase in *Muribaculaceae* (*Bacteriodetes*) and *Enterobacteriaceae* (*Proteobacteria*) during the early phase of LR. These changes normalized during the later phases of LR. Notably, previous studies have shown that GM in patients with non‐alcoholic fatty liver disease (NAFLD) is characterized by a decrease in *Firmicutes* and an increase in *Proteobacteria*, accompanied by reduced species richness [95, 96]. As discussed by Yin et al. [83], this finding is functionally relevant, as *Firmicutes* and *Bacteroidetes* are major contributors to the fermentation of dietary fiber into SCFAs, in contrast to *Proteobacteria*. Therefore, a reduction in fermentation‐competent bacteria may result in decreased availability of microbially derived metabolites that are essential for hepatic lipogenesis, growth, and proliferation.

In this context, it is noteworthy that the study of Sun et al. shows that, at 6 h post‐surgery, the combined relative abundance of *Firmicutes* and *Bacteroidetes* is higher compared to *Proteobacteria* relative to baseline (time 0). This shift may enhance the production of SCFAs and other metabolites, thereby supporting energy supply during the early phases of LR. The correlation between GM and GM‐derived metabolites showed that several metabolic pathways were changed during LR, including pathways in host‐specific metabolism, GM‐specific metabolism, and host and GM co‐metabolism.

Metabolomic profiling analysis revealed time‐dependent changes in various metabolic pathways, including glycolysis, TCA cycle, arginine metabolism, glutathione metabolism, TRP metabolism, and purine and pyrimidine metabolism, all of which are essential for LR. Since these pathways are fundamental to both host and microbial physiology, identifying the exact origin of the metabolites proved challenging. However, the authors suggested that microbial‐host isozymes (microbial enzymes with functions similar to those of host enzymes) may contribute to intestinal metabolism and overall metabolic balance during LR [7].

Correlation analysis identified steroid hormone biosynthesis as the most significantly altered host‐specific pathway during LR. Additionally, BA metabolism, particularly PBA biosynthesis, was notably enriched, indicating a crucial role for cholesterol metabolism in LR. Among GM‐specific pathways, aminobenzoate degradation emerged as particularly important. This pathway, which modulates both aerobic and anaerobic catabolism of aromatic compounds, likely enhances microbial fitness in the gut under fluctuating oxygen conditions [97, 98]. These findings represent the first thorough explanation of the GM‐derived metabolites and how they relate to changes in the GM during LR after 2/3 PH.

Parallel to these findings, Kaur et al. published new evidence in the *American Journal of Physiology—Gastrointestinal and Liver Physiology* [90], where they conducted a metabolomic analysis of peripheral and portal blood from hepatectomized rats, with or without 3 weeks of Abx pre‐treatment, to identify GM‐derived metabolites involved in LR (Figure 3B). Samples were collected 2 days after surgery, coinciding with the peak hepatocyte proliferation [99]. Results showed that hepatectomized rats had an increase in *Bacteroidetes* and *Firmicutes* compared to non‐operated controls. In addition, Abx‐treated rats exhibited reduced LR which was associated to reduced specific phyla, including *Firmicutes*, *Actinobacteria*, and *Bacteroidetes*, supporting the notion that a healthy GM is essential for effective LR [15, 26]. Metabolomic profiling revealed significant changes in both portal and peripheral serum following 2/3 PH in rats. Notably, several metabolites showed opposite trends when comparing portal and peripheral serum: compounds elevated in portal blood (e.g., DCA, propionyl carnitine, certain dipeptides) were reduced peripherally, whereas sphingosine‐1‐phosphate (S1P), which was downregulated in the portal vein, was elevated in the periphery. This compartment‐specific regulation suggests a dynamic redistribution or differential metabolism of key signaling molecules during early LR [90]. Abx treatment before PH further altered these profiles, especially in peripheral serum. Metabolites such as nitrendipine, clofibric acid, 7‐deoxypancratistatin, and N‐phenylacetylglutamine were decreased, while others, including propionyl carnitine, androsterone sulfate, midodrine, fructoselysine, and tricosanedioic acid, were increased. These shifts were suggested to reflect the loss of microbial contributions to host metabolism or the accumulation of metabolites typically processed by the GM [90]. In portal serum, 70 metabolites were commonly altered in both PH and Abx + PH groups. Of these, 52 were increased after PH but were significantly reduced following Abx treatment, including BAs and lipid‐related metabolites such as tetradecanedioic acid, DCA, and linoleyl carnitine. As stated by the authors these patterns indicate that many of the metabolic changes associated with LR are at least partially GM‐dependent. In addition, the inverse regulation of key metabolites like S1P, a signaling lipid involved in cell proliferation [100, 101], and BAs between serum compartments, and their dependence on microbial activity, points to a gut–liver axis that may modulate regenerative processes through BA signaling, lipid metabolism, and microbial‐derived compounds [90]. This aligns with previous evidence linking transient hepatic fat accumulation and BA signaling to hepatocyte proliferation [102]. Kaur et al. [90] also investigated the two key gut‐derived metabolites S1P and DCA in LR by in vitro studies. Results showed that S1P did not stimulate primary rat hepatocyte proliferation. In contrast, DCA at physiological concentrations enhanced proliferation in both human and rodent hepatocytes which was associated to TGR5 and Phosphoinositide 3‐kinase (PI3K)/Protein kinase B (Akt) pathway activation. These results indicated that DCA promotes proliferation via the TGR5‐PI3K‐AKT axis following 2/3 PH, linking microbial metabolism to LR signaling.

Recent studies by Cohen et al. [91] have provided compelling evidence linking GM composition to hepatic regenerative capacity following PH. Cohen et al. examined how distinct GM communities influence intrahepatic microbiome dynamics and post‐PH LR in mice (Figure 3C). After 2 weeks of broad‐spectrum Abx treatment, different groups of mice were recolonized with four microbiota sources—stools from naïve or obese donors, a defined probiotic mixture, or saline as a vehicle control, 5 days prior to surgery. Comprehensive profiling of hepatic bacterial DNA, metagenomic functional pathways, and extracellular vesicle (EV) markers (LPS specific for Gram‐negative bacteria and lipoteichoic acid, LTA, specific for Gram‐positive bacteria) revealed dynamic shifts in intrahepatic microbial load and composition during LR. EVs represent an alternative form by which gut‐derived microbial particles can reach the liver, which can be derived either from bacteria or originate from immune cells. Such EVs were shown to influence hepatic metabolic functions [103]. The relationship between gut and liver microbiota and their combined influence on regenerative capacity was analyzed by comparing stool and liver samples. The outcome of this comparison suggests a synchronized gut‐EV flux. Notably, the abundance of LTA‐positive vesicles dropped sharply immediately after PH, significantly reducing the hepatic LTA/LPS ratio, before returning to baseline in both liver and fecal samples 3 days after surgery.

These findings are consistent with a previous study [103] showing that probiotic‐derived LTA‐positive vesicles enhanced neutrophil and macrophage clearance from resected liver by reducing LPS‐induced VCAM‐1 expression on liver sinusoidal endothelial cells. This LTA‐mediated immune clearance following the first regeneration phases was suggested to support LR and full liver recovery [103, 104].

Consistently, LR was markedly impaired in mice colonized with obese microbiota and reduced LR was linked to a high abundance of intrahepatic Gram‐positive bacteria, potentially serving as a PHLF predictor [91]. Enrichment of aerobic, motile, spore‐forming bacteria and butyrate producers was also negatively correlated with regenerative efficiency, suggesting that specific bacterial functions may hinder hepatocyte proliferation or immune homeostasis during tissue repair. Notably, some bacterial functions were exclusively detected in the liver, including non‐acetate and non‐butyrate producers, as well as aerobic bacteria, particularly in the obese group. These findings suggest that the hepatic microbiome is not merely a direct mirror of the GM but may additionally derive from the portal vein, bile duct, or Kupffer cell‐associated bacterial DNA.

Aggressive microbiome depletion by Abx impaired survival in naïve mice but had minimal impact in obese mice, indicating that host‐microbiota metabolic interactions influence resilience to microbial perturbation. Following PH, a distinct intrahepatic microbial profile emerged between groups. Naïve mice showed intrahepatic dominance of *Enterobacteriales*, *Bacteroidales*, and *Erysipelotrichales*, whereas probiotic‐ and vehicle‐treated groups were enriched in *Clostridiales*. In contrast, obese microbiota‐colonized mice lacked dominant taxa and instead displayed balanced proportions of *Bacillales*, *Lactobacillales*, and *Sphingomonadales*. Although the optimal GM composition for effective LR remains unclear, Cohen et al. [91] demonstrated that a low‐diversity community can still support robust regeneration. In their model, the probiotic‐treated group, dominated by *Firmicutes*, exhibited complete LR after PH, challenging the assumption that higher microbial diversity necessarily reflects a healthier microbiome. The spontaneous migration of gut‐derived bacterial EVs to the liver post‐PH further implicates these vesicles in regenerative signaling. Moreover, the successful outcome in the probiotic group, associated with gut colonization by beneficial Gram‐positive bacteria, further supports the proposed role of LTA‐positive EVs in post‐surgical liver repair [103]. Notably, permitting microbiome recovery after Abx depletion significantly improved survival compared with immediate surgery, indicating that even partial recolonization, whether spontaneous or transplant‐mediated, enhances regenerative capacity. In contrast, GM depletion or dysbiosis impaired LR and promoted the expansion of opportunistic bacteria, while obese‐donor microbiota enriched rare hepatic genera and further reduced LR.

#### 2.1.2 Specific Bacteria and Metabolites Involved in LR

To elucidate the precise contribution of the GM to LR, and to identify the specific microbial taxa and molecular mechanisms that regulate hepatic regenerative responses after PH, these relationships, Guo et al. [92] recently performed 16S rRNA sequencing of fecal samples from mice subjected to PH (Figure 3D). Results showed a distinct postoperative shift in GM composition. In particular, dynamic changes in the relative abundance of *Parabacterioides* (*P*.) *distasonis* were observed in the feces of hepatectomized mice, consistent with the hepatocyte proliferation kinetics. Accordingly, treatment with live *P. distasonis* significantly promoted hepatocyte proliferation and LR after 2/3 PH. Targeted metabolomics revealed a significant positive correlation between *P. distasonis* and β‐hydroxybutyric acid (BHB), as well as hyodeoxycholic acid and 3‐hydroxyphenylacetic acid in the gut content after surgery. However, only treatment with BHB significantly promoted hepatocyte proliferation and LR in mice after 2/3 PH. Mechanistically, *P. distasonis* boosted FA oxidation and increased BHB production in the liver, which promoted LR after surgery via activation of STAT3 signaling. Consequently, pharmacological STAT3 inhibition attenuated the BHB‐mediated promotion of cell proliferation and LR both in vitro and in vivo. Previous studies have shown that BHB reduces hepatocellular necrosis in mouse models of liver ischemia–reperfusion injury and alcoholic liver disease [105, 106, 107]. This evidence further confirms the role of BHB in alleviating liver injury during LR after 2/3 PH in mice.

These findings provide new insights into the potential of selected microbial species as therapeutic targets to enhance hepatic regeneration after PH. A beneficial effect on LR attributable to a specific bacterium was also recently reported by Hu et al. [93] in metabolic dysfunction‐associated fatty liver disease (MAFLD) (Figure 3E). MAFLD is an increasingly prevalent chronic liver condition that progresses from simple steatosis to steatohepatitis, fibrosis, cirrhosis, and HCC. Importantly, hepatic steatosis is associated with impaired LR and poorer postoperative outcomes following PH [29, 30, 108, 109, 110]. Therefore, identifying therapeutic strategies to improve LR in MAFLD patients is of significant clinical relevance. Growing evidence indicates that MAFLD progression is closely linked to GM alterations [111, 112, 113]. To better understand the role of GM during LR in MAFLD, Hu et al. [93] employed a combination of GM and liver metabolomics on hepatectomized (50% PH) mice with High Fat Diet (HFD)‐induced or Choline‐deficient (CD)‐HFD‐induced MAFLD. These studies revealed that PH induces marked but transient changes in GM composition, with a general trend toward recovery over time. Early after surgery, an increase in *Escherichia–Shigella* was observed, consistent with its known association with inflammatory responses. In parallel, transient increases in *Muribaculaceae*, *Bacteroides*, and *Akkermansia* were detected, taxa previously implicated in LR by regulating lipid metabolism [83, 114, 115, 116]. Among these, *Akkermansia*, particularly *Akkermansia muciniphila* ( *A. muciniphila* ), emerged as a key bacterium. Its abundance increased at the onset of LR and peaked around day 3 after PH, coinciding with the maximum regenerative phase. Moreover, its levels positively correlated with the rate of LR. This is notable because *Akkermansia* is typically reduced in MAFLD [117] and *A. muciniphila* is known to exert beneficial effects in MAFLD by improving gut barrier integrity and modulating the gut–liver axis [114]. However, its role and mechanisms in LR remain unknown. To determine whether *A. muciniphila* directly contributes to LR, MAFLD mice were treated with this bacterium prior to surgery. The treatment not only attenuated MAFLD severity, evidenced by reduced body and liver weight gain, as well as lower triglyceride and alanine aminotransferases (ALT) levels, but also significantly enhanced hepatocyte proliferation after PH. Similar results were obtained in *A. muciniphila* ‐treated germ‐free mice, supporting a direct effect independent of other microbial interactions. Further metabolomic analyses suggested a potential mechanism underlying these effects. *Akkermansia* abundance was positively associated with metabolites involved in the TCA cycle, a central pathway responsible for ATP production and the generation of biosynthetic intermediates required for rapid cell growth [118]. This finding indicates that *A. muciniphila* may promote LR by enhancing hepatic energy metabolism. In summary, *A. muciniphila* appears to ameliorate MAFLD and to accelerate LR following PH, potentially by inducing metabolic reprogramming. However, it remains to be clarified whether its pro‐regenerative effects are independent of its ability to reduce hepatic steatosis.

#### 2.1.3 Dietary Factors Promoting LR by GM Regulation

Several factors regulate the GM and thereby influence LR. As the gut is the primary site of nutrient digestion and absorption, dietary factors play a crucial role in shaping microbial composition [32]. Malnutrition can promote the translocation of gut bacteria and their metabolites [119], whereas certain dietary components with anti‐inflammatory or antioxidant properties help maintain microbial balance [120]. Diets enriched with protein, vitamins, or fish oil have been shown to alleviate hepatic ischemia–reperfusion injury, while folic acid and vitamins ameliorate alcoholic liver disease [121].

Maintaining adequate vitamin D levels appears to play an important role in modulating GM composition. As summarized by Tangestani et al. [122], vitamin D supplementation is associated with an increase in *Bacteroidetes* and a reduction in *Firmicutes*, along with changes at the genus level, including members of the *Lachnospiraceae* family (e.g., *Blautia, Roseburia, Dorea*, and *Coprococcus*). The biological effects of vitamin D are mediated by the vitamin D receptor (VDR), and increasing evidence supports a role for the VDR axis in hepatic pathophysiology [123, 124, 125]. Impaired vitamin D signaling has been linked to increased fibrosis across multiple tissues, including the liver [123, 125, 126, 127, 128], as demonstrated by VDR knockout models that develop spontaneous fibrosis and cirrhosis [125]. VDR activity in hepatic stellate cells (HSCs) regulates the TGF‐β signaling pathway, shifting its effects from profibrotic toward regenerative responses [125], while hepatocytes express low basal levels of VDR [129]. To examine whether VDR in hepatocytes contributes to recovery from liver injury, Elangovan et al. [94] investigated LR after 2/3 PH in hepatocyte‐specific VDR‐null (hVDR KO) mice (Figure 3F). Hepatocyte‐specific VDR deletion impaired LR, as evidenced by reduced hepatocyte proliferation and marked increases in circulating conjugated BAs [94]. Since serum BAs mainly reflect enterohepatically recirculated BAs that are not efficiently cleared by the liver, this finding suggests persistent hepatic dysfunction in hVDR KO mice throughout the evaluated time points. In addition, hVDR KO mice showed reduced levels CDCA, a potent FXR agonist known to promote LR [130], which may have further contributed to the impaired regenerative response [94]. Reverse transcription‐polimerase chain reaction (RT‐ PCR) analysis revealed significant alterations in the expression of cell‐cycle genes in hVDR KO livers. Consistent with the role of VDR as a transcription factor acting through interaction with retinoid X receptor (RXR) [131, 132], gene expression profiling of vitamin D‐induced changes demonstrated that VDR regulates pathways involved in cell proliferation, survival, oxidative stress, and acute‐phase responses in hepatocytes [94]. Interestingly, under basal conditions, hVDR KO mice exhibited only minimal changes in genes related to oxidative stress and acute‐phase responses. This finding is in line with the low basal expression of VDR in unstressed hepatocytes and suggests that VDR may play a particularly important role during recovery from hepatic injury.

Overall, the available evidence highlights the GM as a central regulator of LR after PH, acting through tightly coordinated metabolic, immunological, and signaling interactions within the gut–liver axis. Regeneration was consistently associated with time‐dependent shifts in microbial composition and function that paralleled the different phases of hepatic repair, indicating a synchronized process of host‐microbiome metabolic remodeling. In the early postoperative period, transient changes in the relative abundance of *Firmicutes*, *Bacteroidetes*, and *Proteobacteria* reshaped pathways involved in energy production, amino acid turnover, and BA metabolism, thereby supporting the increased bioenergetic and biosynthetic demands required for hepatocyte proliferation. These dynamic microbial adaptations were not merely correlative, as antibiotic‐induced depletion of the GM disrupted BA homeostasis, impaired hepatocellular proliferation, and ultimately compromised regenerative capacity, underscoring the microbiome's essential role in maintaining a permissive regenerative environment.

A central mechanistic link emerging from these studies involves microbiota‐derived metabolites and signaling mediators. Among these, DCA was identified as a key pro‐regenerative metabolite capable of promoting hepatocyte proliferation through activation of the TGR5‐PI3K‐AKT pathway, thereby directly connecting microbial BA metabolism with hepatic proliferative signaling. At the same time, EVs, particularly LTA‐positive vesicles, functioned as critical intermediaries between intestinal microbial activity and hepatic repair processes, highlighting that microbiome‐driven regulation of LR extends beyond bacterial abundance alone to include microbial‐derived signaling particles. Interestingly, several bacterial functions were identified exclusively within the liver rather than in the gut, supporting the concept that the liver represents a distinct microbial and metabolic niche shaped by, but not entirely reflective of, intestinal microbial composition. Together, these findings suggest that LR depends on an integrated network in which microbial metabolites, EVs, and BA‐mediated signaling cooperate to orchestrate hepatic recovery.

The therapeutic relevance of this gut–liver crosstalk is further reinforced by studies demonstrating that restoration of microbiome homeostasis, whether spontaneous or probiotic‐mediated, improves both survival and regenerative outcomes after surgery. In this context, beneficial bacterial taxa such as *P. distasonis* and *A. muciniphila* emerged as potential microbiota‐based therapeutic candidates capable of enhancing hepatic recovery. However, the regenerative effects of these organisms appear to be strongly influenced by the underlying hepatic environment. Guo et al. investigated LR in healthy livers, whereas Hu et al. examined regeneration in animals with chronic liver injury and MASL, conditions known to impair regeneration through persistent inflammation, fibrosis, and epithelial dysfunction [133]. Consequently, while the effects observed for *P. distasonis* may have broader physiological relevance, the benefits attributed to *A. muciniphila* may currently be more specific to diseased metabolic contexts. This distinction emphasizes that the impact of microbiome modulation on LR is likely dependent on baseline hepatic health and disease‐associated alterations in host‐microbial interactions.

Within this framework, vitamin D status may represent an additional regulatory layer linking host metabolism, fibrosis, and microbial homeostasis. Vitamin D deficiency may impair LR not only directly, by reducing hepatocyte proliferative capacity and promoting hepatic stellate cell‐mediated fibrogenesis [125, 134, 135], but also indirectly through disruption of GM composition and gut–liver metabolic crosstalk. Since vitamin D is an important modulator of intestinal microbial ecology, deficiency‐related dysbiosis could exacerbate the inflammatory and metabolic disturbances that impair LR. Thus, vitamin D signaling, microbial composition, BA metabolism, and hepatic proliferative pathways appear to converge within an interconnected regulatory network controlling post‐PH repair.

Collectively, these findings position the gut–liver axis as a dynamic and integrated regulator of post‐PH regeneration, in which microbial composition, microbial‐derived metabolites, EV‐mediated communication, host metabolic status, and hepatic pathological context jointly determine regenerative outcomes. This integrated model not only advances the mechanistic understanding of LR but also highlights the translational potential of GM‐targeted therapies, probiotic supplementation, BA signaling modulation, EV‐based interventions, and metabolic optimization strategies as novel approaches to improve hepatic recovery after surgery.

### 2.2 GM and LR After Liver Resection: Human Studies

**FIGURE 4:**
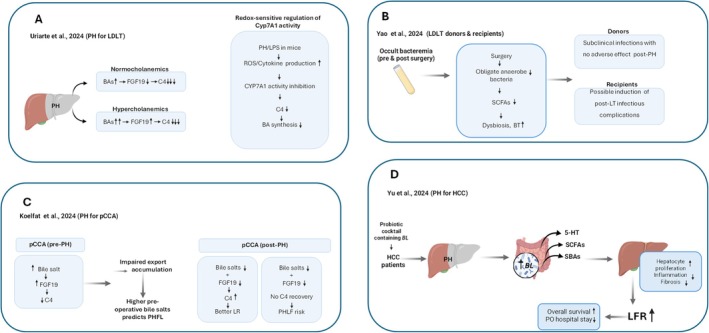
GM and LR after liver transplantation and liver tumors: human studies. (A) Uriarte et al. [136]: serum BAs increase after PH. In patients undergoing PH for LDLT with pronounced hypercholanemia, FGF19 was upregulated in contrast to the decreased FGF‐19 levels observed in normocholanemic controls. Preoperative C4 levels were significantly higher in hypercholanemic patients, but both groups (hyper‐ and normo‐cholanemic) quickly reached similarly low levels within 2 h after PH. Experimental studies in hepatectomized/LPS treated mice supported CYP7A1 regulation through post‐transcriptional and redox‐dependent post‐translational pathways. BAs, bile acids; C4, 7α‐hydroxy‐4‐cholesten‐3‐one; CYP7A1, cholesterol 7α‐hydroxylase; FGF19, fibroblast growth factor 19; LDLT, living‐donor liver transplant; LPS, lipopolysaccharide; PH, partial hepatectomy. (B) Yao et al. [137]: occult bacteremia was detected in both LDLT recipients and healthy donors before and after surgery. Following surgery, both donors and recipients exhibited a reduction in POAs, which are major producers of SCFAs. Decreased SCFA production may contribute to intestinal dysbiosis, impaired gut barrier integrity, and increased BT. In healthy donors, intact immune function likely limits bacterial load and virulence, resulting in subclinical bacteremia. In contrast, in immunosuppressed recipients, occult bacteremia may increase the risk of progression to overt post‐transplant infection. BT, bacterial translocation; LDLT, living‐donor liver transplant; POAs, predominant obligate anaerobes; SCFAs, short chain fatty acids. (C) Koelfat et al. [138]: PCCA patients showed preoperative adaptation of the bile salt–FGF19 axis, with elevated bile salts and FGF19 levels and suppressed CYP7A1‐mediated synthesis. High preoperative bile salt acids predicted PHLF. Unlike controls, PH does not further increase circulating bile salts in pCCA patients, although the preserved inverse relationship between FGF19 and C4 indicates intact gut–liver crosstalk despite altered biliary anatomy. Restoration of C4 levels to control values by postoperative day 7 suggested recovery of bile salt synthesis and was associated with effective LR, whereas patients who developed PHLF failed to restore C4 levels after surgery. C4, 7α‐hydroxy‐4‐cholesten‐3‐one; CYP7A1, cholesterol 7α‐hydroxylase; FGF19, fibroblast growth factor 19; LR, liver regeneration; pCCA, perihilar cholangiocarcinoma; PH, partial hepatectomy; PHFL, post‐hepatectomy liver failure. (D) Yu et al. [139]: administration of a probiotic cocktail containing *BL* to patients with HCC undergoing PH throughout the perioperative period improved PO recovery, reduced hospital stay, and increased 1‐year survival. These benefits were likely mediated by reduced inflammation and fibrosis, along with enhanced hepatocyte proliferation, potentially through modulation of 5‐HT, SBAs, and SCFA pathways. 5‐HT, 5‐hydroxytryptamine; *BL*, *Bifidobacterium longum* ; HCC, hepatocarcinoma; LFR, liver function recovery; PH, partial hepatectomy; PO, postoperative; SBAs, secondary bile acids; SCFAs, short chain fatty acids.

Alterations in the GM might be responsible for the post‐operative outcomes in different conditions which require the presence of a relationship between the intestine and the liver. Such is the case with the clinical surgical procedures of hepatic resections in the context of LDLT or tumor removal (see Figure 4).

#### 2.2.1 LR in the Context of LDLT

LT is an effective treatment for end‐stage liver disease, with significant improvements in outcomes for patients with cirrhosis and HCC. However, despite advancements in surgical techniques and immunosuppressive therapies, extending post‐transplant survival and reducing perioperative and postoperative complications remain significant challenges in LT [140]. Common postoperative complications include acute and chronic rejection, infections, bleeding, and biliary‐related issues [141].

Since its introduction in 1963 [142], the demand for liver grafts has steadily increased, resulting in significant organ shortages, long waiting lists, and increased dropout risk. To address the gap between supply and demand, alternative strategies such as living donor liver transplantation (LDLT) have been adopted [143, 144, 145]. Unlike deceased donor liver transplantation (DDLT) grafts, which can undergo prolonged periods of cold storage, LDLT grafts are typically implanted within hours of procurement, therefore minimizing the ischemia–reperfusion injury associated with prolonged graft preservation, which is a well‐known cause of early graft dysfunction and is negatively associated with post‐transplant regeneration and survival [146, 147]. The quality of the graft is superior in LDLT due to the rigorous selection process. Living donors are rigorously screened healthy individuals, providing steatosis‐free grafts with optimal functional hepatic mass [148]. In contrast, DDLT grafts often come from extended‐criteria donors with comorbidities that may compromise graft quality. LDLT also enables transplantation at lower Model for End‐Stage Liver Disease (MELD) scores under optimized clinical conditions, avoiding deterioration associated with prolonged waiting times [149, 150, 151]. These factors minimize preservation injury, optimize graft function, and improve recipient health, likely contributing to the survival benefit reported by Rashid et al. [152]. However, donor PH is not without risk. A recent meta‐analysis of over 60 000 living donors reported an overall morbidity rate of 25%, with major complications in 5.5% and a mortality rate of 0.06% [153]. While definitive superiority remains unproven and requires more robust comparative studies, current evidence supports LDLT as an important option for patients requiring LT [152]. Its broader application should be considered, particularly in regions with a severely limited deceased donor pool. However, concerns regarding the risks associated with the LDLT procedure may have hindered its wider adoption in Western countries, underscoring the need for continued efforts to ensure donor safety and preserve quality of life following donation. In this context, further studies aimed at better characterizing LR outcomes in both LDLT recipients and donors are urgently needed.

As previously discussed, maintaining BA balance during LR is critical to prevent liver damage and support growth.

Uriarte et al. [136] recently investigated the early response of the BA‐FGF19 axis in healthy individuals undergoing PH for LDLT (Figure 4A). In the same study, the authors also evaluated BA synthesis in mice following PH and acute inflammation, focusing on the regulation of CYP7A1, the rate‐limiting enzyme in BA synthesis from cholesterol. Consistent with previous reports, circulating BA levels increased rapidly after surgery in LDLT healthy donors, likely reflecting the reduced residual liver mass and its limited ability to process the portal BA load [62, 64, 154, 155, 156]. Importantly, a subgroup of hypercholanemic patients exhibited a substantially greater increase in serum BAs than normocholanemic patients [136]. In these individuals, BA accumulation was mainly driven by PBAs, suggesting impaired bacterial BA biotransformation consistent with a degree of cholestasis. Differences were also observed in FGF19 dynamics. Normocholanemic patients showed an early and progressive decline in circulating FGF19 after PH, whereas hypercholanemic patients exhibited a rapid and significant FGF19 increase at very early postoperative timepoints, a finding not previously reported [154]. Experimental studies have highlighted a critical role for FGF19/FGF15 during LR, not only by protecting against BA toxicity but also by directly promoting LR independently of BA signaling [59, 60, 68]. However, the precise behavior and role of FGF19 during human LR remain poorly understood. Under physiological conditions, FGF19 is primarily produced by ileal enterocytes and released into the portal and systemic circulation in response to BA flux [157, 158, 159]. Nevertheless, hepatic FGF19 expression has also been documented during cholestasis, suggesting that hepatocytes may become an alternative source under BA overload conditions [160, 161, 162, 163, 164]. Although Uriarte et al. could not determine the precise origin of circulating FGF19, several findings supported a hepatic origin in hypercholanemic patients including: (i) BA levels comparable to cholestatic conditions associated with documented hepatic FGF19 expression [160]; (ii) the ability of primary human hepatocytes to induce FGF19 expression in response to BAs within the range observed in these patients [165, 166]; and (iii) reduced bacterial transformation of PBAs implying decreased intestinal BA flux and limited ileal FXR‐dependent FGF19 secretion [167]. These findings further support the concept that early alterations in enterohepatic BA circulation after PH may significantly affect BA signaling. This is particularly relevant considering the pivotal role of the GM in BA metabolism and in the regulation of LR [34, 83].

Suppression of CYP7A1‐mediated BA synthesis is a recognized hepatoprotective response following PH [59, 68, 168, 169]. CYP7A1 activity can be indirectly assessed by serum levels of 7α‐hydroxy‐4‐cholesten‐3‐one (C4), a stable downstream metabolite [170, 171]. Uriarte et al. [136], found that preoperative C4 levels were significantly higher in hypercholanemic patients, although both patient groups rapidly reached similarly low levels within 2 h after PH. These findings suggest that some healthy individuals may naturally exhibit a higher basal rate of BA synthesis. The reasons underlying baseline variability in C4 concentrations remain unclear, although associations with body weight and sex have been described [172]. Notably, the rapid postoperative decline in C4 in LDLT donors exceeded expectations based on the extent of liver resection and the known reduction in Cyp7A1 mRNA expression (approximately 40% within 2 h in humans) [154]. This observation suggests the existence of additional post‐transcriptional or post‐translational mechanisms inhibiting CYP7A1 activity after surgery. The authors also observed markedly reduced C4 levels in septic patients which gradually normalized during recovery, indicating profound suppression of BA synthesis during acute systemic stress.

Experimental studies further supported early inhibition of CYP7A1 activity after PH and during inflammatory stress conditions: (i) intrahepatic C4 levels declined in both hepatectomized and sham‐operated mice before detectable changes in CYP7A1 protein, characterized by a low turnover, suggesting surgery‐ or stress‐related signals independent of liver mass reduction; (ii) circulating C4 levels also decreased under acute inflammatory conditions induced by LPS treatment before the reduction in CYP7A1 protein due to the marked reduction in *CYP7A1* levels became evident; and (iii) the LPS‐induced reduction in C4 was attenuated by an antioxidant‐enriched diet, implicating redox‐dependent regulation.

Overall, these observations support the existence of post‐translational mechanisms regulating CYP7A1 activity and BA synthesis independently of CYP7A1/Cyp7a1 transcription. These findings are physiologically significant because sustained CYP7A1 suppression profoundly affects lipid and glucose metabolism and is essential for maintaining cholesterol homeostasis [58, 173, 174, 175, 176]. Preservation of hepatic cholesterol is critical not only for immune defense during infection [174, 177, 178] but also for supporting the rapid cellular proliferation required for LR [179, 180, 181]. Accordingly, redox‐sensitive regulation of CYP7A1 enzymatic activity [182] may represent a mechanism to balance BA synthesis, cholesterol availability, and regenerative demands.

Bacteremia is a major complication after LDLT and adversely affects patient outcomes [183, 184]. Although bacterial translocation (BT)–the passage of bacteria or bacterial products from the gut lumen to extraintestinal sites–is well recognized, the predominant bacterial strains and their relationship with the GM remain poorly understood. In a recent prospective study, Yao et al. [137] simultaneously analyzed blood and fecal samples from 20 LDLT recipients and their donors, using rRNA‐targeted quantitative RT‐PCR (RT‐qPCR), to detect occult bacteremia, defined as a subclinical/asymptomatic bloodstream infection not able to be detected by conventional culture methods [137] (Figure 4B). Remarkably, occult bacteremia was found in both recipients and healthy donors at similar frequencies before and after surgery. These findings suggested that BT may occur even in healthy individuals under transient physiological stress that compromises intestinal barrier integrity (“leaky gut”) [185]. In healthy donors intact immunity likely was suggested to keep bacterial load and virulence low, resulting in subclinical bacteremia. In contrast, in immunosuppressed recipients occult bacteremia could predispose to post‐LT progression to overt infection. Male sex and low body mass index (BMI) were associated with occult bacteremia [137], consistent with known sex‐related differences in immune responses [186] and the link between low BMI, sarcopenia, and reduced infection resistance [187, 188, 189]. Notably, patients with occult bacteremia, both donors and recipients, showed fewer predominant obligate anaerobes (POAs) after surgery, key producers of SCFAs [190, 191]. Reduced SCFA production might contribute to intestinal dysbiosis, impaired barrier function, and enhanced BT [192, 193, 194]. However, since some patients had reduced POAs, while others did not, even after the same surgery, specific predispositions might exist. Notably, the bacterial species detected in blood were derived from the GM but were not necessarily those overgrowing in the intestine. Authors suggested that occult bacteremia and overt infection are, therefore, distinct phenomena: occult bacteremia may represent a transient physiological event caused by various low‐virulence bacteria, whereas clinically apparent infections are typically driven by classical pathogens with higher virulence potential [195, 196, 197].

Despite its limited sample size, the Yao et al. study provides the first longitudinal evidence of occult bacteremia during LDLT. Continuous molecular monitoring could help identify at‐risk patients and guide infection control.

#### 2.2.2 LR After Perihilar Cholangiocarcinoma (pCCA) and Liver Resection

Perihilar cholangiocarcinoma (pCCA) is a rare biliary tract malignancy in which extensive liver resection remains the only curative option. However, postoperative mortality remains high (reported in up to 19% of patients) and is most often caused by PHLF [198, 199, 200, 201]. A central driver of this risk is the disruption of bile salt homeostasis. While bile salts act as essential signaling molecules, excessive intrahepatic accumulation is hepatotoxic, inducing apoptosis and necrosis [202, 203]. Under cholestatic conditions, bile salt synthesis is normally suppressed through hepatic FGF19 production, which likely regulates hepatic BA production via auto‐ or para‐crine signaling pathways [160, 204, 205, 206, 207]. However, how this regulatory process works in the regenerating (post)cholestatic liver after surgery is not well understood, with only limited data available from humans [65, 162, 208].

Koelfat et al. recently filled this gap by studying perioperative bile salt‐FGF19 signaling in patients with pCCA, comparing them with non‐cholestatic controls undergoing resection for colorectal liver metastases (CRLM) [138] (Figure 4C). The strength of this study is its pre‐post design with PH and repeated postoperative sampling, enabling detailed assessment of the bile salt‐FGF19 axis and bile salt synthesis. The data from this study demonstrate a preoperatively adapted gut–liver crosstalk via the bile salt‐FGF19 axis in patients with pCCA, characterized by increased systemic bile salt and FGF19 levels and near‐complete suppression of CYP7A1‐mediated bile salt synthesis (indicated by low C4 levels). Bile salt accumulation in pCCA was likely due to impaired bile salt export caused by decreased bile salt export pump (BSEP) expression [209] or accumulation within the dilated biliary tree [210]. Importantly, elevated preoperative intrahepatic bile salt levels predicted postoperative hyperbilirubinemia and were highest in patients who subsequently developed PHLF, underscoring the hepatotoxic potential of bile salt retention. Unlike non‐cholestatic controls, PH in pCCA was not associated with postoperative increases in circulating bile salts. Postoperative FGF19 levels were lower than those in both CRLM controls and healthy volunteers [211] although the inverse relationship between FGF19 and C4 was maintained, suggesting an intact gut–liver crosstalk despite altered biliary anatomy. Notably, C4 levels returned to control values by day 7 post operation, a novel finding in human patients, indicating restoration of bile salt synthesis within the first week. Notably, individuals who developed PHLF failed to restore C4, indicating that the resumption of bile salt synthesis may serve as a marker of metabolic reserve and regenerative capacity.

#### 2.2.3 HCC, LR, and GM

HCC accounts for approximately 90% of primary liver cancer cases and remains the third leading cause of cancer‐related mortality worldwide. Liver resection is among the most important therapeutic strategies for patients with HCC and plays a key role in extending long‐term survival [212, 213, 214, 215, 216, 217]. However, despite its clinical relevance, the rate of liver function recovery (LFR) after hepatectomy and its impact on prognosis are not well defined. The speed and degree of postoperative LFR are vital for surgical safety, as insufficient recovery can lead to postoperative liver failure and poor long‐term outcomes. Existing evidence suggests that LFR is affected by several factors, including preoperative liver reserve function, residual liver volume, and underlying liver disease [218, 219, 220, 221, 222]. Thus, identifying and validating mechanisms that promote LFR is of great clinical value.

In this context, Yu et al. [139] provided critical translational insights by demonstrating a central role for the GM, particularly for *Bifidobacterium longum* (*BL*), in promoting LFR after hepatectomy in HCC patients (Figure 4D). Using both retrospective and prospective cohorts, they showed that LFR status was strongly associated with overall survival (OS). In a retrospective cohort of 490 patients, those with normal recovery (NR) had significantly better three‐year OS compared to those with delayed recovery (DR). This association was confirmed in a prospective cohort of 123 patients, where LFR status correlated with two‐year OS. Importantly, patients with DR exhibited distinct alterations in GM composition compared to those with NR within 5 days after surgery, and *BL* emerged as a key microbial species linked to favorable LFR. Preclinical experiments further confirmed the causal role of *BL*. Oral supplementation of *BL* improved LFR in mice that received fecal microbiota transplants from DR patients. Moreover, in a clinical trial, administration of a probiotic cocktail containing *BL* significantly increased the proportion of patients achieving NR, reduced hospitalization time, and improved overall survival. Mechanistically, Yu et al. [139] identified several pathways by which *BL* supplementation improved liver recovery. First, metabolic pathway enrichment analyses showed increased activity of the TRP biosynthesis pathway in *BL*‐treated patients. This was accompanied by significantly higher levels of 5‐hydroxytryptamine (5‐HT) in both fecal and serum samples. Since 5‐HT is primarily produced by intestinal enterochromaffin cells and can also be generated by certain gut microbes [223, 224, 225], its elevation suggests a gut–liver axis in LFR. Functionally, 5‐HT is known to stimulate hepatocyte proliferation by activating 5‐HT receptors, thereby promoting DNA synthesis and cell cycle progression [226, 227, 228, 229]. In line with this mechanism, *BL*‐treated patients exhibited increased hepatocyte proliferation markers such as cyclin D1 and Ki67. Second, *BL* supplementation significantly altered BA metabolism. As *BL* can convert PBAs into SBAs, changes in BA composition were observed in *BL*‐treated patients. Although excessive SBA accumulation has been linked to liver dysfunction [230, 231, 232, 233], the *BL*‐associated changes in this study correlated with reduced liver fibrosis and inflammation, suggesting that restoration of BA homeostasis may contribute to improved recovery. Third, *BL* treatment increased the level of the SCFA butyrate, a microbial metabolite with anti‐inflammatory properties. While *Bifidobacteria* typically produce acetate and lactate depending on substrate availability [234], cross‐feeding interactions within the GM can boost butyrate production. Consistent with previous studies, elevated SCFAs likely contributed to the observed reduction in liver inflammation [235, 236].

Collectively, the studies by Uriarte et al., Yao et al., Koelfat et al., and Yu et al. provide converging evidence that LR post PH or LT is profoundly shaped by dynamic interactions within the gut–liver axis, particularly through coordinated regulation of BA metabolism, GM composition, intestinal barrier integrity, and microbial‐derived signaling pathways. Together, these findings support a model in which postoperative hepatic adaptation depends not only on intrinsic regenerative mechanisms but also on the maintenance of metabolic and microbial homeostasis during the acute regenerative phase.

Uriarte et al. demonstrated that LDLT donors who developed postoperative hypercholanemia exhibited marked alterations in BA and FGF19 signaling, characterized by elevated circulating BA and FGF19 levels together with suppression of CYP7A1‐mediated BA synthesis. These findings suggest the activation of a rapid hepatoprotective feedback mechanism aimed at limiting further BA accumulation during acute hepatic stress and regeneration. Importantly, the study proposed that impaired bacterial BA biotransformation and altered enterohepatic BA circulation contribute to this dysregulation, thereby linking postoperative microbial alterations directly to BA‐mediated regenerative signaling. In addition, the observation that CYP7A1 activity may be regulated through rapid post‐transcriptional or post‐translational mechanisms, potentially involving redox‐sensitive pathways, highlights a previously under‐recognized level of metabolic control during LR.

The mechanistic relevance of this adaptive BA‐FGF19 response is reinforced by the findings of Koelfat et al. in pCCA patients undergoing PH. Similar to the hypercholanemic subgroup described by Uriarte et al., these patients displayed profound BA accumulation, markedly elevated FGF19 levels, and severe suppression of CYP7A1 activity pre‐operatively, reflected by reduced C4 concentrations. Importantly, recovery of BA synthetic capacity during the first postoperative week occurred only in patients with preserved liver function, whereas individuals who developed PHLF failed to restore CYP7A1 activity. Integrating both studies suggests that transient suppression of BA synthesis represents an early adaptive and hepatoprotective response to BA overload, whereas persistent dysregulation of the BA‐FGF19‐CYP7A1 axis may indicate impaired hepatic metabolic reserve and defective regenerative competence. Thus, restoration of CYP7A1‐mediated BA synthesis may serve not only as a marker of metabolic recovery but also as an indicator of successful LR.

In parallel, Yao et al. extended the understanding of postoperative gut–liver interactions by providing the first longitudinal evidence of occult bacteremia in both LDLT recipients and healthy donors. Their findings linked postoperative bacterial translocation to alterations in GM composition, particularly reductions in SCFA‐POA, suggesting that disruption of GM homeostasis and barrier integrity occurs early after surgery. These observations complement the metabolic findings of Uriarte et al. by indicating that impaired microbial composition may simultaneously alter BA transformation, weaken intestinal barrier function, and facilitate BT, thereby amplifying systemic inflammatory and metabolic stress during LR. Together, these studies support the concept that postoperative disturbances in BA metabolism, microbial ecology, and intestinal permeability are closely interconnected components of the regenerative response.

The translational relevance of these mechanisms is further emphasized by the work of Yu et al., who demonstrated that perioperative GM modulation through *B. longum* supplementation enhanced LFR in HCC patients undergoing PH. The beneficial effects were associated with improvements in SCFA production, BA metabolism, and 5‐HT‐related signaling pathways, ultimately resulting in shorter hospitalization and improved postoperative survival. These findings integrate closely with those of Uriarte, Yao, and Koelfat by suggesting that preservation or restoration of microbial homeostasis may help stabilize BA signaling, maintain intestinal barrier integrity, and optimize regenerative metabolic pathways during the postoperative period. In particular, the restoration of SCFA‐producing bacteria may counteract the microbial depletion and barrier dysfunction observed after surgery, while modulation of BA metabolism may help prevent persistent dysregulation of the BA‐FGF19 axis associated with impaired LR and postoperative liver dysfunction.

Taken together, these studies support an integrated model in which PH, LDLT, or cholestatic injury induces acute disturbances in BA homeostasis, GM composition, and intestinal barrier function, leading to compensatory activation of BA‐FGF19 signaling and suppression of CYP7A1‐mediated BA synthesis as an early hepatoprotective response. While transient activation of this pathway may facilitate adaptation to hepatic stress, persistent disruption of BA synthesis, microbial homeostasis, and intestinal integrity appears to contribute to impaired LR and postoperative complications, including PHLF. Conversely, therapeutic restoration of microbial balance through probiotic or microbiota‐targeted strategies may enhance regenerative competence by simultaneously modulating BA metabolism, SCFA production, intestinal permeability, and inflammatory signaling. Overall, these findings position the gut microbiome‐BA‐FGF19 axis as a central integrative regulator of hepatic recovery and highlight its potential as a therapeutic target for improving outcomes after liver surgery and transplantation.

## 3 Conclusions and Future Directions

This synthesis highlights the central role of the GM and its metabolites as dynamic regulators of LR following PH and LT. The evidence supports a model in which modulation of GM composition and function offers a promising therapeutic approach to enhance postoperative recovery and reduce complications such as PHLF. A key implication is the potential for dietary and probiotic interventions to rebalance dysbiotic microbial communities and restore essential metabolic pathways. Studies consistently show that beneficial taxa, such as *A. muciniphila* , *B. longum* , and *P. distasonis* , promote LR by modulating pathways including the TCA cycle, SCFA production, and BA metabolism. These findings suggest that targeted nutritional strategies or probiotic supplementation could enhance hepatocyte proliferation and reduce inflammation, particularly in metabolically compromised conditions such as MAFLD. In this context, precision GM modulation tailored to a patient's metabolic status may represent a next‐generation perioperative strategy.

Beyond compositional changes, microbial‐derived molecules, including metabolites, bacterial DNA, and EVs, emerge as key mediators of gut–liver communication. The translocation of these components to the liver after surgery suggests a functional signaling axis that can either promote or impair regeneration. Notably, the balance between LPS and LTA may serve as a surrogate marker of microbial influence, with the LPS/LTA ratio representing a promising predictive biomarker for PHLF. Integration of such markers into clinical monitoring could enable early risk stratification and personalized intervention. Therapeutically, EV‐based interventions may provide a means to selectively deliver beneficial microbial signals while avoiding the risks associated with live bacteria.

The role of host‐microbiome metabolic crosstalk is particularly evident in BA signaling. The FXR‐FGF19 axis serves as a key adaptive pathway in response to BA overload after PH, protecting hepatocytes and supporting regeneration. This underscores the therapeutic potential of FXR agonists and FGF19 mimetics to mitigate BA‐induced toxicity and reduce PHLF risk. Notably, adjunctive therapies such as vitamin D supplementation may further enhance LR through host signaling pathways, particularly via the VDR, which regulates cell‐cycle progression and BA homeostasis. This supports the concept that optimal postoperative recovery relies on coordinated modulation of both microbial and host factors.

In the context of LT, detecting occult bacteremia highlights the complexity of host‐microbiome interactions in immunocompromised patients. Although often subclinical, its association with increased post‐LT infections indicates that ongoing microbiological monitoring may enhance patient management. Further research is necessary to clarify causal relationships and assess whether GM‐targeted interventions can reduce these risks.

Clinical translation will require well‐designed trials to validate probiotic formulations, microbial metabolites, and targeted therapies across diverse patient populations. A multimodal approach that integrates microbiota modulation, metabolic monitoring, and targeted pharmacologic interventions may ultimately offer the most effective strategy to improve LR and surgical outcomes.

## Author Contributions

Review concept and design (M.P.), illustration preparation (M.P.), data collection (M.P.), data interpretation (M.P., R.L.), and drafting and revision of the manuscript (M.P., R.L., A.C., and G.S.). All authors have made a significant contribution to this paper and have approved the final text.

## Funding

The authors have nothing to report.

## Conflicts of Interest

The authors declare no conflicts of interest.

## Data Availability

No new data.
